# Physical Unclonable Function based on a Multi-Mode Optical Waveguide

**DOI:** 10.1038/s41598-018-28008-6

**Published:** 2018-06-25

**Authors:** Charis Mesaritakis, Marialena Akriotou, Alexandros Kapsalis, Evangelos Grivas, Charidimos Chaintoutis, Thomas Nikas, Dimitris Syvridis

**Affiliations:** 1 Eulambia Advanced Technologies Ltd. Ag. Ioannou 24, 15342 Athens, Greece; 2 Department of Informatics & Telecommunications, National and Kapodistrian University of Athens, Panepistimiopolis Ilisia, 15784 Athens, Greece

## Abstract

Physical unclonable functions are the physical equivalent of one-way mathematical transformations that, upon external excitation, can generate irreversible responses. Exceeding their mathematical counterparts, their inherent physical complexity renders them resilient to cloning and reverse engineering. When these features are combined with their time-invariant and deterministic operation, the necessity to store the responses (keys) in non-volatile means can be alleviated. This pivotal feature, makes them critical components for a wide range of cryptographic-authentication applications, where sensitive data storage is restricted. In this work, a physical unclonable function based on a single optical waveguide is experimentally and numerically validated. The system’s responses consist of speckle-like images that stem from mode-mixing and scattering events of multiple guided transverse modes. The proposed configuration enables the system’s response to be simultaneously governed by multiple physical scrambling mechanisms, thus offering a radical performance enhancement in terms of physical unclonability compared to conventional optical implementations. Additional features like physical re-configurability, render our scheme suitable for demanding authentication applications.

## Introduction

**Figure 1:**
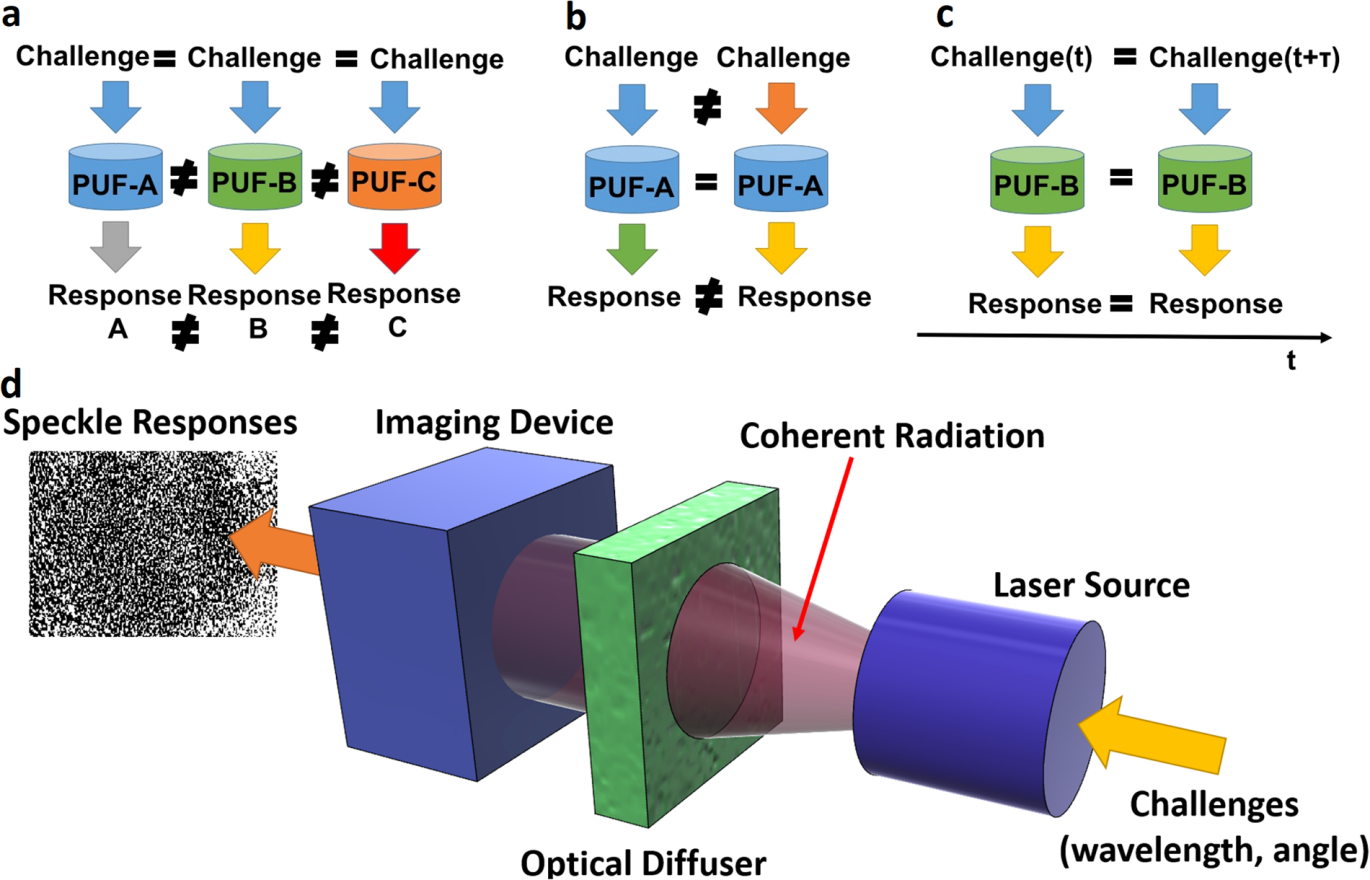
Basic PUF properties: (**a**) unclonability output depends on the physical properties of the PUF, (**b**) unpredictability the output depends on the input, (**c**) time-invariant operation (robustness) (**d**) schematic of a typical optical PUF based on an optical diffuser.

Physical unclonable functions (PUFs) have received considerable attention, due to unique security features related to their physical complexity^1^. Briefly, PUFs employ the use of disordered physical objects or random processes, which can generate unpredictable outputs (responses) under extrinsic excitation (challenges). The physical processes governing the behaviour of such systems are so complex that they cannot be reliably reverse - engineered by either computational or physical techniques. However, the interaction between the applied stimulus and the physical object employed is purely deterministic^1^, meaning that in the case the same physical object – stimulus combination is employed, the same response will be generated (Fig. 1a–c).

Especially, in an internet-of-things (IoT) ecosystem, where the devices are densely interconnected, bearing minimum security features due to hardware limitations, the absence of any cryptographic sensitive data (private keys etc.) in the deployed system is a significant security leverage. Based on these properties, PUFs have already infiltrate a wide range of applications^2^, including cryptographic key generation^3,4^, software-hardware interconnection^5^, authentication tokens^6,7^, whereas PUFs have also shielded systems against code-reuse attacks^8^.

So far, the spotlight of attention has been mainly focused on silicon - cast PUFs, whose principle of operation is based on exploiting uncontrollable variations in operational parameters^2–7,9^. Existing implementations include ring-oscillators^9–11^, arbiter PUFs^12,13^, static random access memory (SRAM) PUFs^14,15^, resistive random access memory (RRAMs)^16,17^, XOR-Arbiters^18^, and imaging CMOS PUFs^19,20^. Despite their merits in terms of integration^21^, unclonability, and robustness^22–24^, the underlying physical scrambling mechanism, in most cases, is rather simplistic, resulting to enhanced vulnerability to modelling attacks^25–27^. The arsenal of adversaries is further enhanced through a plethora of side-channel attacks^28–30^. Newly emerging PUF implementations based on nanofabrication procedures^31–35^, hold great promise, but current results are mainly focused on providing proof of concept and do not evaluate their cryptographic performance.

Optical PUF’s physical mechanism relies on the random interference pattern (speckle) created when a laser beam propagates through an inhomogeneous material (Fig. 1d). The corresponding existing schemes, employ transparent tokens containing randomly micro-structures^1,36^, laser-engraved samples^37^, or sheets of regular paper^38^. Their security is based on the complexity of the underlying physical mechanism where a modelling attack would require the division of the token into wavelength sized voxels and solving Maxwell’s equations for each possible arrangement^36^. This physical complexity, renders optical PUFs more secure than their electronic counterparts. On the other hand, the majority of optical implementations suffer from increased footprint and are considered vulnerable to machine learning based attacks. Under this attack methodology, an adversary gains control of the raw PUF’s responses and the corresponding challenges, and exploits these pairs so as to gain information about the transfer function (TF) of the system. In the vast majority of optical PUFs, this vulnerability stems from the linearity of the scattering process^36^.

Within this research landscape, we put forward an alternative optical PUF configuration based on an optical waveguide as the PUF’s sole physical token. The structural parameters of the proposed PUF are chosen so as to enable the excitation and efficient guiding of a high number of transverse modes. The proposed configuration’s physical scrambling mechanism, in addition to scattering, includes sensitive multi-mode interference mechanisms and in-fiber propagation related impairments. These features offer enhanced structural sensitivity and thus lower probability of physical cloning, whereas, at the same time, allow physical re-configurability. Furthermore, the proposed PUF’s principle of operation, involves multiple interfaces that render their physical replication significantly more challenging compared to conventional optical approaches. These pivotal advantages, combined with similar device’s cost and fabrication requirements with a conventional optical PUF, render our scheme an attractive solution. Therefore, the proposed scheme can replace conventional optical PUFs in applications that range from random number generation to authentication^39^. In this work, we focus on the latter scenario, assuming honest and malicious manufacturers’ attempts to physically clone the device. A twofold investigation is used that consists of experimental evaluation, highlighting the role of the multiple scrambling mechanisms and a numerical model to confirm the operational principle.

## Theoretical Background

**Figure 2:**
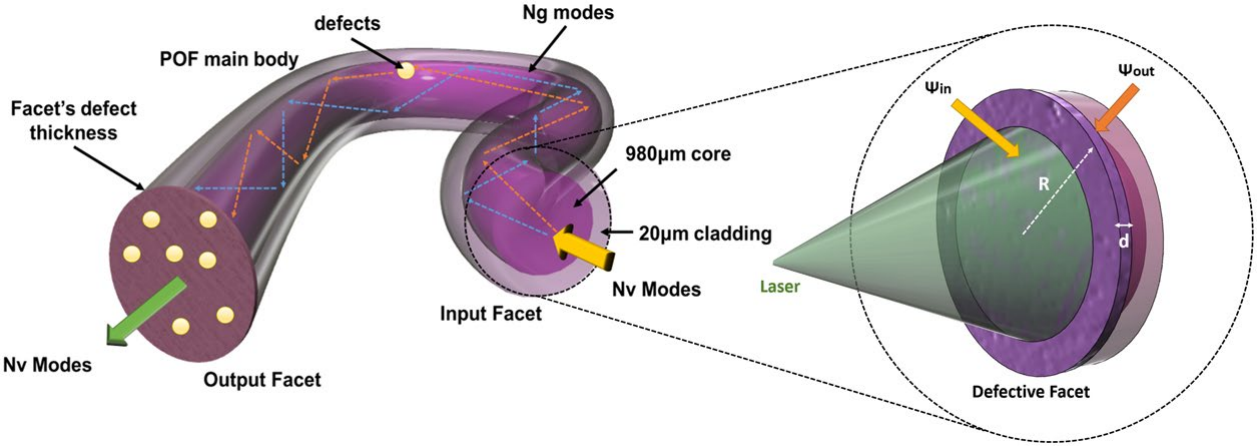
Schematic of the proposed PUF implementation, presenting all physical mechanisms associated with response generation. Inset depicting the friction processed fiber’s facet, R and d correspond to the radius of the facet and to the average defect size. Ψ_in_ and Ψ_out_ are related to the initial excited and filtered modes, due to the core-cladding of the fiber.

The core of the proposed PUF implementation is an optical waveguide or, in our case, a polymer optical fiber (POF) (Fig. 2) able to facilitate an extensive number of transverse optical modes. The POF specimen acts simultaneously as an optical waveguide and as an optical scattering token. From an operational point of view, the PUF token can be divided into three virtual sections. The first section comprises the fiber’s input (facet) which, in general, contains a random number of structural defects (scratches, scattering centres, impurities, refractive index anomalies etc.). These defects result from intentional processes, like noise-driven mechanical friction, and are combined with unintentional random effects imposed during manufacturing.

Assuming that the fiber’s input surface exhibits structural defects with thickness (d), larger than the wavelength of illumination (λ) and shorter than the mean free path of photons in the material (λ«d«l), we can treat the fiber’s input as a typical optical diffuser^40^ (Fig. 2). Therefore, its statistical properties can be mathematically formulated as an assembly of random photon walks. Under this assumption, theory dictates that ${N}_{v}=\frac{2\pi {\rm A}}{{\lambda }^{2}}$ independent modes (Ψ_in_) can be invoked, depending on the fiber’s facet area (A) (${N}_{v}\approx 2.05\cdot {10}^{6}$ neglecting polarization states, assuming a circular area with diameter of 1 mm and λ = 1550 nm)^40,41^. Due to the limited thickness (losses due to propagation), the majority of these modes will not be attenuated and will carry energy at the other side of the facet (Ψ_out_), which in our case is the core-cladding interface (Fig. 2). In principle, each outgoing transverse mode decomposes to a linear combination of all the incoming modes following: ${\Psi }_{out}^{j}=\sum _{i=1}^{N}{T}_{i,j}\cdot {\Psi }_{in}^{i}$, where T_i,j_ is the scattering matrix of the defective fiber’s input^40,41^. In an ideal case, this diffuser’s TF contains ${N}_{V}\cdot {N}_{V}$ elements, that correspond to the physical complexity of this section. Even partial knowledge of these can enable PUF’s TF extraction^42^. Nonetheless, in the proposed PUF, the modes’ spatial distribution cannot be thoroughly monitored, as in a typical optical PUF, even using sophisticated techniques^43,44^. Furthermore, these initial modes will interact with the boundary conditions imposed by the fiber’s core-cladding interface; therefore, a number of modes will not be supported, due to the limited numerical aperture (NA). This mode-filtering process results in an upper limit for the transverse guided modes (Ψ_g_) that is governed by N_g_ = (π∙R∙NA/λ)^2^, where R is linked to the radius of the fiber^45^; using the above parameters (R = 0.5 mm, NA = 0.5, λ = 1540 nm), ${N}_{g} < 2.5\cdot {10}^{5}$. Therefore, the facet-fiber interface results to a mode filtering by 87% compared to a thin optical diffuser, where the vast majority of modes exit the material. The aforementioned formula provides an upper bound regarding the fiber supported modes, nonetheless the exact number of modes that will be excited and their inter-modal power distribution is directly linked to the illumination conditions. Therefore, we can assume that if the laser parameters remain constant (wavelength, angle, focus, etc.), the initial modal distribution is governed by the facets’ defects and the fiber’s boundary conditions.

The second operational section consists of the main body of the optical fiber, in which, supported transverse modes (Ψ_g_) will propagate sharing the same wavelength but exhibiting fixed group velocity differences^45^. The fiber propagation is anticipated to affect the initial modal-distribution by re-scrambling modal power or by exciting new transverse modes. The underlying physical mechanism, can be attributed to mechanical deformations or common in-fiber defects which are byproducts of the manufacturing process, like refractive-index variations (Rayleigh scattering), or micro-cracks (Mie scattering) (Fig. 2). Overall, these effects will modify each mode’s spatial profile but will also induce mode-coupling effects^45^. Contrary to typical PUF implementations, these in-fiber effects cannot be thoroughly monitored or controlled, whereas accurate measurements of the complex field (Ψ_g_) is not feasible without irrevocably altering the physical structure of the specimen^43,44^. The length of the PUF should remain small so as not to enhance these deficiencies and force the initial inter-modal distribution to converge to a power equilibrium^45^. The third operational section of the proposed PUF is the fiber’s output that, similarly to the input, has a random number of wavelength-sized or larger defects, able to scatter the guided optical modes. The fiber’s output is illuminated by the guided optical field, so Ν_v_ “incoming” modes are excited and these modes will allow N_v_ output modes (Ψ_out_). Therefore, the last operational section of our scheme acts similarly to a typical diffuser, allowing Ψ_in_ modes from the internal of the fiber structure to evoke Ψ_out_ modes, whose number is governed again only by the area of the fiber’s surface. Therefore, the last operational section of the PUF re-expands the number of transverse modes (Ν_ν_) to a number similar to a typical thin optical diffuser. Based on the above, the PUF’s overall transfer matrix has a size of N_v_xN_v_, which in turn depends on the waveguide’s cross-section and input wavelength, while its rank is governed by N_g._

A typical operational scenario includes a laser source illuminating the fiber’s facet; the structural defects at the facet that act as miniaturized deflectors, feeding the fiber with a random initial mode distribution. The modes propagate inside the fiber, exchanging power due to internal imperfections and illuminate the output facet, which acts as a second diffuser. It is clear that, following the above description, the speckle pattern observed after the output facet, is considered the system’s response and is simultaneously governed by all PUF’s virtual sections. The proposed PUF is an extension of conventional optical approaches, and thus can be used under similar operational modes, nonetheless here we focus on the physical uniqueness of our device that acts as an authentication token.

## Experimental Setup

**Figure 3:**
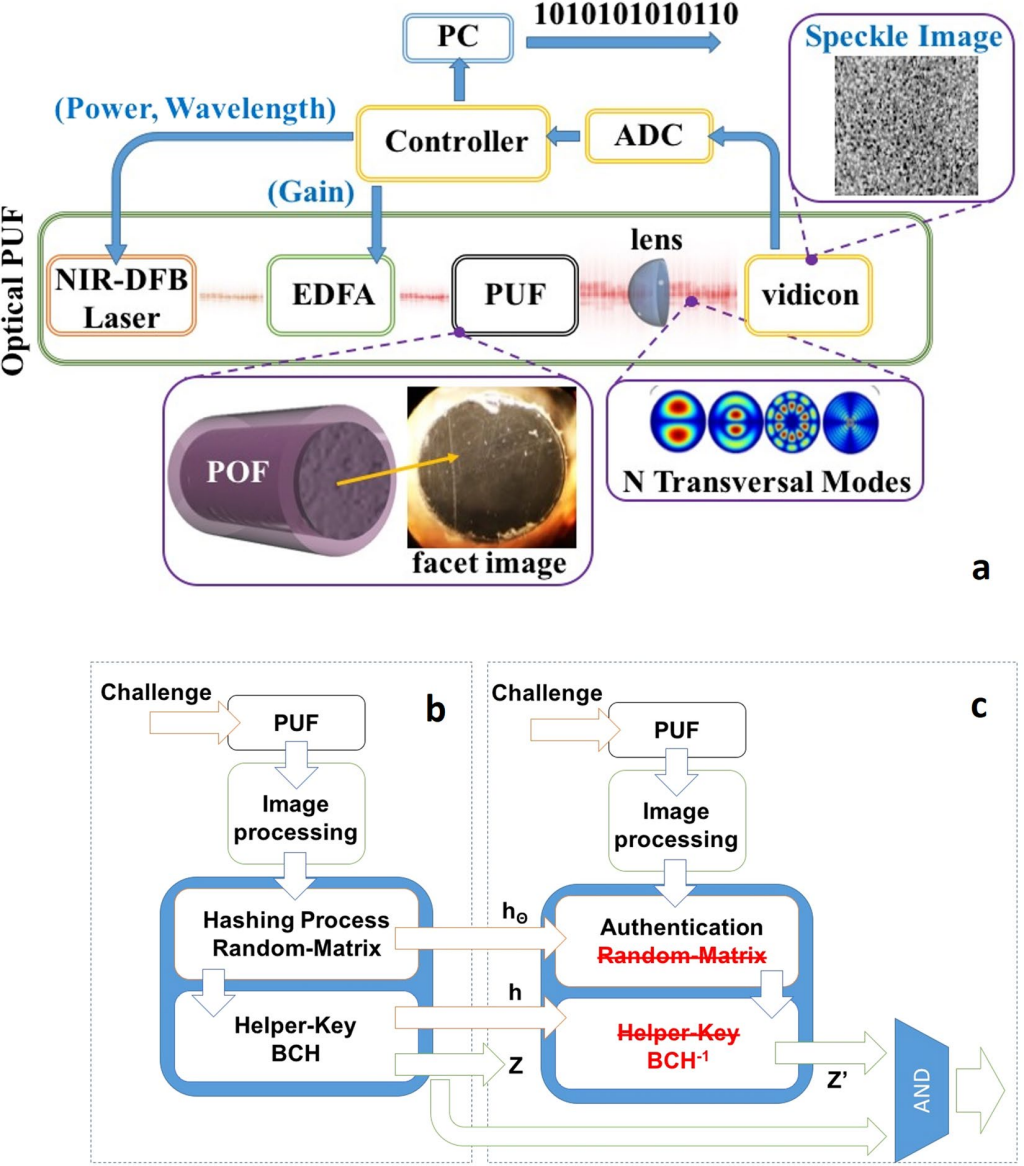
(**a**) Block diagram of the experimental setup, using bulk optics. NIR-DFB corresponds to near infrared distributed feedback laser, EDFA to erbium doped fiber amplifier, POF stands for polymer optical fiber. In the insets from left-to-right: microscope image of the fiber’s processed facet followed by a typical speckle image acquired. Block diagram of the (**b**) Key-generation procedure used in^37^ and (**c**) Key authentication.

The experimental configuration is presented in Fig. 3a (methods). At its core, it consists of a short piece of commercial large-core polymer optical fiber as the PUF specimen. The fiber’s facets exhibit random defects (inset of Fig. 3a), whereas optical challenges are being generated by a laser. Aiming to amend the detrimental effects of experimental noise and generate time invariant binary strings, the raw output of the PUF is processed through fuzzy extractor techniques^46^ combined with hashing approaches, like the random binary method^47^ or the Gabor binary method^48^. These methodologies have been integrated in a general security framework that encompasses a holistic security analysis^37^. Figure 3b,c demonstrate a brief overview of the process that allows the generation of the binary strings (code-word) and the corresponding helper data (error correction bits). In brief, random pixels are sampled from the speckle-like image and, in the case of random binary hashing, are multiplied with a matrix presenting a random normal distribution and are quantized using the mean image intensity. Similarly, during Gabor hashing, post processing that involves Gabor filters is applied and Gabor coefficients are chosen, instead of raw image pixels (methods). This data can be used for authentication when the PUF is used, as key authenticator, presented in Fig. 3c (methods). A critical factor in the system’s performance evaluation is the overhead imposed by the inclusion of redundant bits (error correction). Therefore, reproducibility (robustness) of the responses and resiliency to cloning are benchmarked versus the bit correcting capabilities^37,49,50^.

Robustness quantifies a system’s resilience to external perturbations and essentially is the probability of generating the same raw response, whenever a single PUF component is repeatedly measured over the same challenge. With respect to the key generation process depicted in Fig. 3b,c, it can be expressed as the conditional probability of producing an identical binary output (z) in both modes, using the helper data created in the setup mode (Fig. 3b). In our case, three different PUF’s instantiations are used; each instant is studied separately under stable laser excitation, where 60 images are acquired, covering a time span of several minutes. Physical unclonability is related to an adversary’s potential to possess two different PUFs that provide the same response under identical excitation conditions and in terms of key generation, it can be expressed as the conditional probability of generating the same binary output (z) from two different components in both modes, using the helper data created in the setup mode. It is, in practice, a measure of the system’s security level against cloning by honest and malicious manufacturers; meaning the unintentional construction of PUFs that provide the same response, while the second type corresponds to the intentional manipulation of the fabrication process so as to produce identical PUFs. Similarly, to previous works^1,34–38^, the case of an honest manufacturer is quantified by employing responses derived by applying the same challenge to PUFs produced through the same fabrication procedure. In this work, the dataset consists of responses from 10^3^ different PUFs, obtained under identical experimental conditions.

## Experimental Results

**Figure 4:**
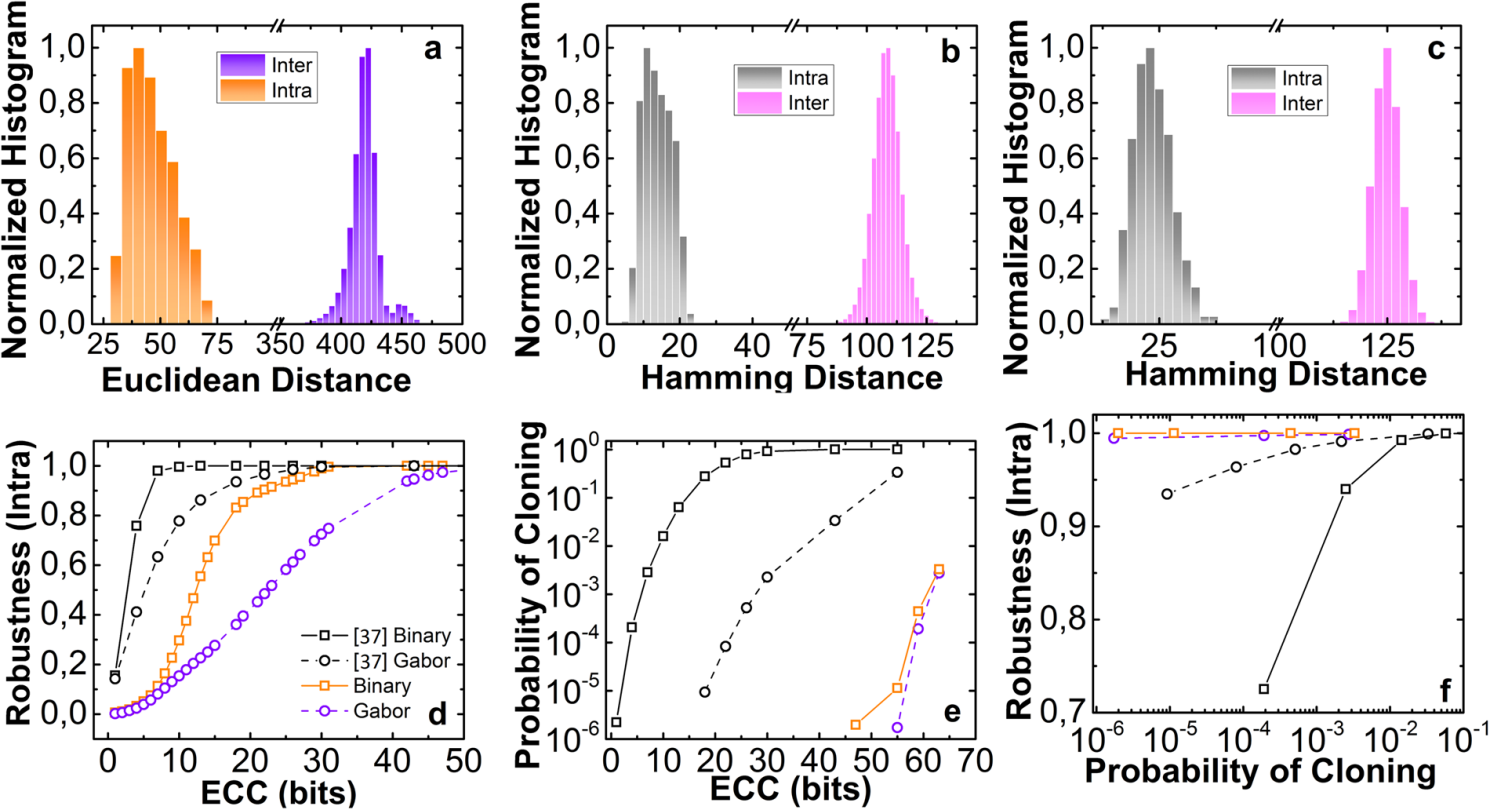
(**a**) Euclidean distances for normalized raw responses (images 8bit, 340 × 340). “Inter” corresponds to images from different PUF instantiations (10^3^) and “intra” to multiple images for the same PUF-challenge combination. (**b**) Hamming distances for pairs of code words generated through random binary hashing and (**c**) through the spatial Gabor filtering. (**d**) Robustness of the proposed system (with Random and Gabor) and^37^ versus the error correction capability (ECC) bits employed. (**e**) Probability of cloning for 255-bit long code-words generated from 10^3^ POF-PUFs and using the data provided in^37^ versus the error correction capabilities. (**d**) Combined graphs presenting Robustness versus the attained probability of cloning for the proposed scheme and^37^.

The first metric for evaluating the system’s efficiency against honest manufacturers is the Euclidean distances between images from each data-set (Fig. 4a). In the case that the robustness dataset is employed (Fig. 4a - intra) we estimate the impact of noise, while if the second set of 1000 different PUF items is used, the variation of the PUF’s response is evaluated (Fig. 4a - inter). The mean value for the intra-distance was computed D_intra_ = 40.5, whereas for the inter case D_inter_ = 422.5. This significant difference, alongside the lack of any overlap between the two distributions, is perquisite for the efficient operation of such a system^1^ because it eradicates the possibility that two different PUF instantiations to be falsely considered the same PUF affected by noise.

The second standard metric is the hamming distance of bit-strings that have been generated by each response using either the Random binary (Fig. 4b) or the Gabor binary hashing technique (Fig. 4c). The generated binary strings have a total length of 255 bits. The Random binary technique is not computationally demanding, while Gabor hashing involves adaptive spatial filtering of the image. Similar to the Euclidean distance metric, the first dataset (intra) provide the magnitude of noise induced bit-flips, whereas the second set (inter) reflects flips due to unintentional fabrication variations. In Fig. 4b can be observed that the number of bit-flips in the intra case has a mean value of 13.7 ± 3.9 (5.3% ± 1%) compared to 107.8 ± 4.9 (42.2% ± 2%) of the inter case. In Fig. 4c where Gabor-hashing has been utilized, intra and inter case mean values have been slightly increased to 25 bit-flips (9.8%) and 125 (49%) respectively. This performance deviation depends on the Gabor coefficient selected during hashing. An important feature is that both hashing methods provide no overlap between the two distributions, thus minimizing the probability of false positives.

The system’s robustness is computed by using the process depicted in Fig. 3b,c. Each PUF-challenge combination from the first dataset (same PUF, same challenge) is used to produce a binary string and the corresponding helper data (Fig. 3b). These outputs are fed in the system that now is set to authentication mode (Fig. 3c) allowing the generation of a new code word (z′). The probability that z ≠ z′ scales with the error correction capabilities. For evaluation purposes in Fig. 4d robustness is computed for the proposed scheme and a typical image-based PUF^37^ versus the error correction capabilities (number of redundant bits) for both hashing techniques. Comparison with^37^ is chosen because it is a typical example of an optical PUF, whereas it is the only case where PUF’s performance is evaluated using a cryptographic framework, instead of Euclidean distance-based metrics.

It can be seen that the typical optical PUF offers increased robustness compared to our scheme. For example, by employing Random binary hashing the typical image-based PUF requires at least 10 error correcting code (ECC) bits to attain a robustness level of 1, while for the proposed scheme similar performance is achieved for at least 30 bits. Although this result weights against our scheme, it is worth mentioning that the same mechanisms trigger an enhancement in PUF’s response diversity. Therefore, for a complete system evaluation both metrics should be considered. Furthermore, a similar trend regarding robustness is seen in the case of Gabor binary hashing.

Employing the same methodology, the second dataset (10^3^ PUFs) is used to estimate the probability of cloning. In this case, a binary key and helper data are generated by each PUF. These data are fed to each of the other PUFs, which are set to authentication mode. Through this methodology, the probability of a false positive is computed versus the error correction capabilities. In Fig. 4e the probability of cloning for our scheme is demonstrated versus the number of ECC bits for both hashing techniques. It is evident that the proposed scheme provides radical performance enhancement that exceeds 10^6^ for a 44 ECCs and Random binary hashing, whereas for the Gabor binary hashing the same trend is preserved. For lower error correction capabilities, the probability of cloning is zero for the proposed scheme, and a substantial higher number of PUF instantiations should be generated so as to compute non-zero values. For comparative reasons in Fig. 4f the probability of cloning versus the robustness is demonstrated for both systems. It is clear that our system vastly outperforms typical approaches preserving robustness, while exhibiting a probability of cloning in the order of 10^−6^. On the other hand, for the case of Gabor binary hashing and for 50 ECC bits our approach allows a reduction of the probability of cloning by 5 orders of magnitude compared to^37^ (Fig. 4f). The performance of Gabor binary hashing in this case as well, is governed by the selected coefficients, thus aiming to benchmark our system with^37^ we preserved the same selection process.

**Figure 5:**
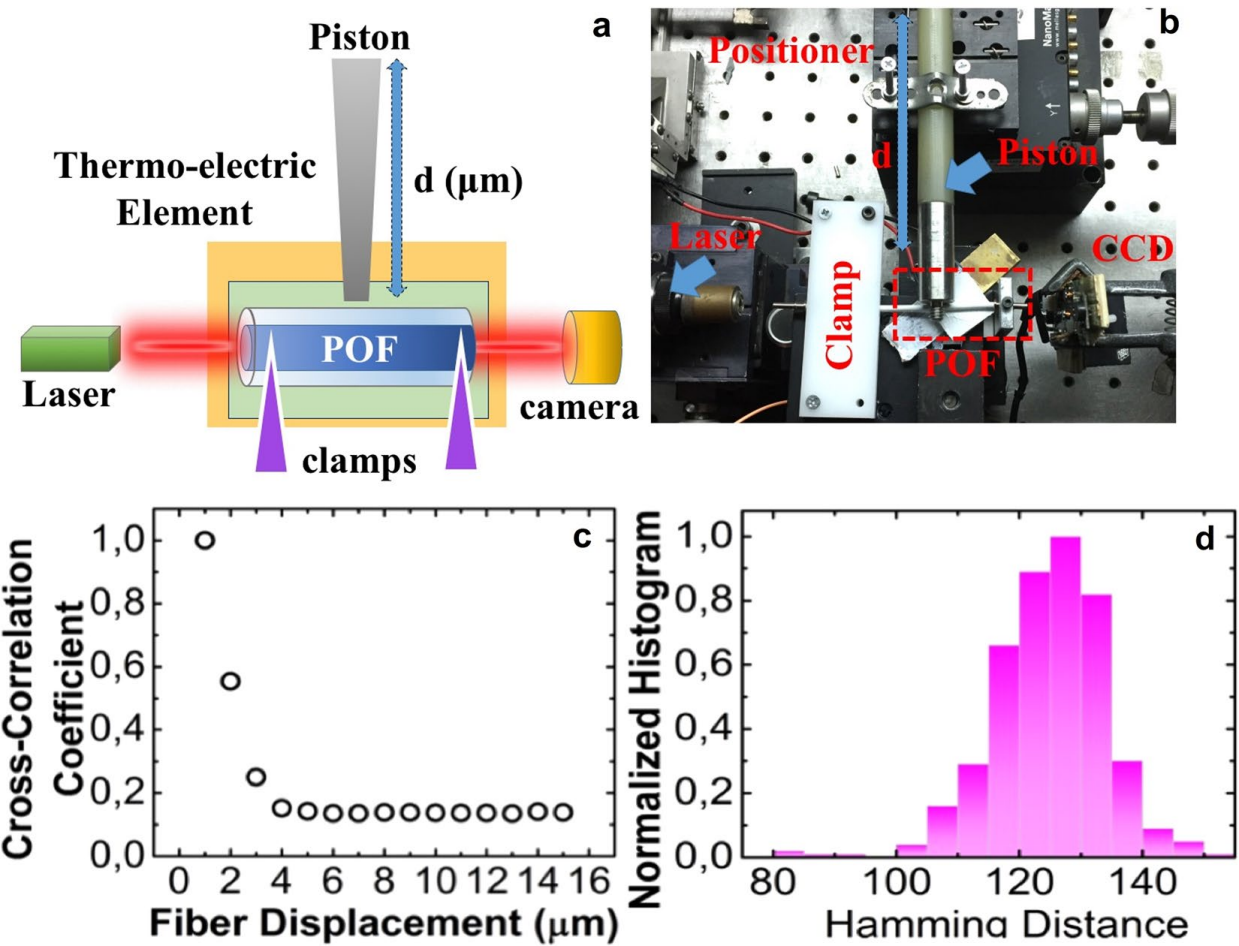
(**a**) Schematic representation of the experimental setup used for testing sensitivity to fiber deformations. (**b**) Experimental image: the system is equipped with a thermoelectric cooler and a piston that can deform the fiber, attached to a NanoMax TS301 micro-positioner. (**c**) Cross correlation of consecutive images versus the fiber deformation (**d**) hamming distances of the generated code-words for the same challenge-PUF under mechanical deformation.

The aforementioned results target the impact of facet’s defects. Aiming to investigate the role of propagation in the PUF’s response, we modified the original setup by including a piston that could apply tension with a micrometre precision at the fiber (Fig. 5a). The two ends of the fiber were fixed (Fig. 5a,b). The applied displacement had a minimum step of 1μm, whereas illumination was constant. The cross-correlation coefficient of each consecutive image compared to the initial has been computed in Fig. 5c. It is evident, that a minor displacement of 3μm de-correlates responses (<0.15). These responses allowed the extraction of code-words and in Fig. 5d the hamming distance of these code-words is computed. It can be seen that a limited number of samples provide a reduced bit-flip probability (31.3%), whereas the vast majority of samples are uncorrelated offering a hamming distance of 127.3 bit ∼ 50%, under a displacement >3 μm.

## Numerical Analysis

Aiming to interpret the aforementioned experimental results, we developed a numerical model with enhanced physical accuracy (methods). Its core is the computation of spatial distributions and phase velocity of all the transverse modes supported in an optical waveguide with similar characteristics to the experimental PUF. Defective facets act as typical diffusers, the fiber’s main body was treated as an ideal medium, whereas output is projected to an imaging device. We assume that the defects at the input provide a unique inter-modal power distribution, thus for each PUF instantiation we assumed the excitation of random modes with normal power distribution. The output facet acts as a diffuser providing random amplitude and phase modulation, following a normal distribution, at each spatial partition.

**Figure 6:**
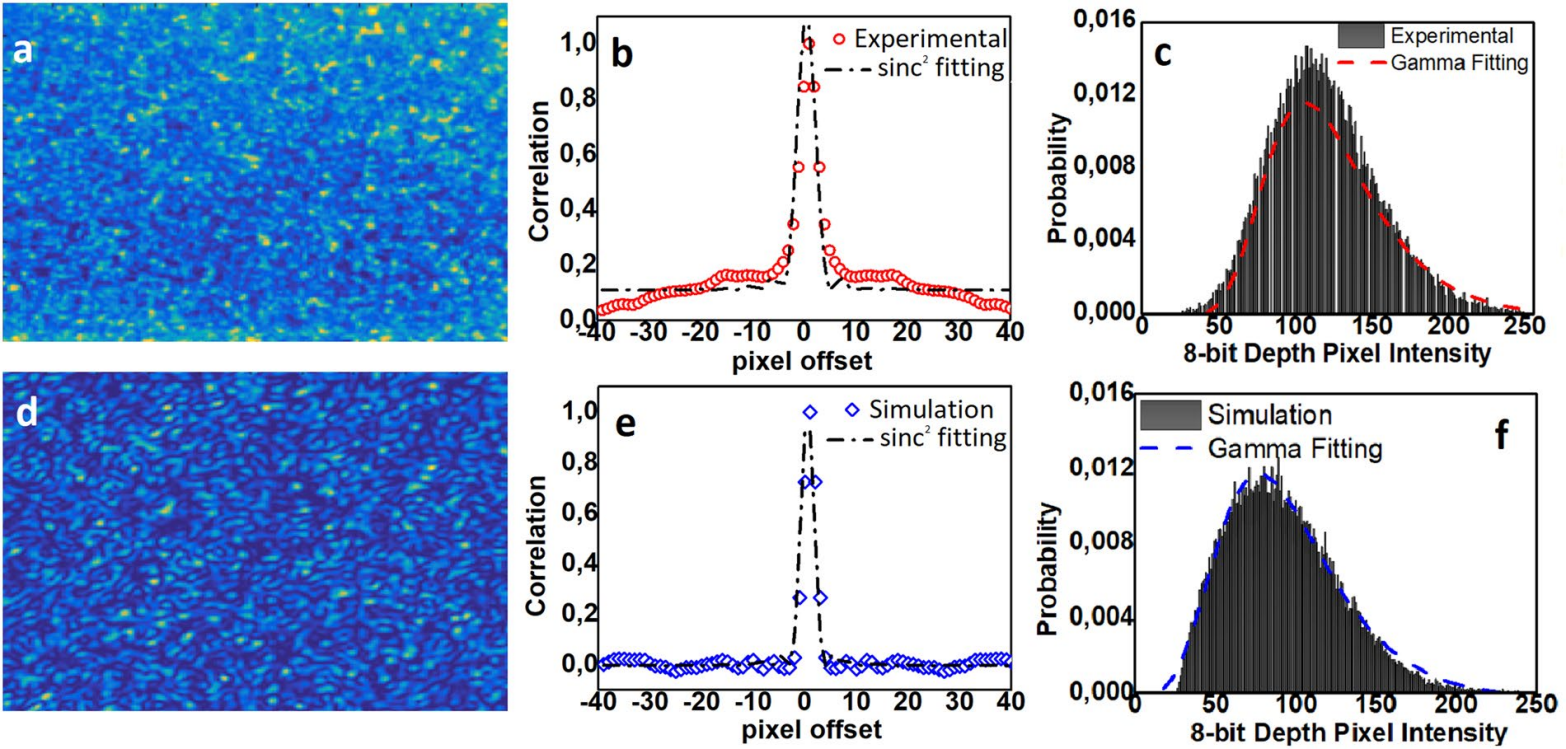
Experimental: (**a**) cropped image of the speckle with dimension of 200 × 200 (**b**) autocorrelation function alongside sinc^2^ fitting providing average speckle size of 6 pixels (**c**) intensity histogram alongside gamma function fitting. Simulation: (**d**) image speckle (**e**) autocorrelation function with d = 6 pixels (**f**) intensity histogram for the simulation data.

Aiming to validate the principle of operation, we used typical experimental images (Fig. 6a) and computed the average speckle size (d = 6 pixels), following a typical experimental-oriented methodology (methods) (Fig. 6b) and their intensity distribution (Fig. 6c). This distribution was fitted assuming a gamma-function, as dictated by^51^. We set the camera-PUF distance (D_x_ = 15 cm) similar to the experimental setup. We assumed that 100 random high-order modes (TE_m,k>100_) exhibiting random power following normal distribution were excited due to input facet’s defects. The emerging speckle was computed (Fig. 6d) and similar metrics were used as before (Fig. 6e,f). It can be seen that the same grain size was computed, whereas intensity followed a gamma-distribution. The experimental deviations occur due to existence of ambient light during measurements.

**Figure 7:**
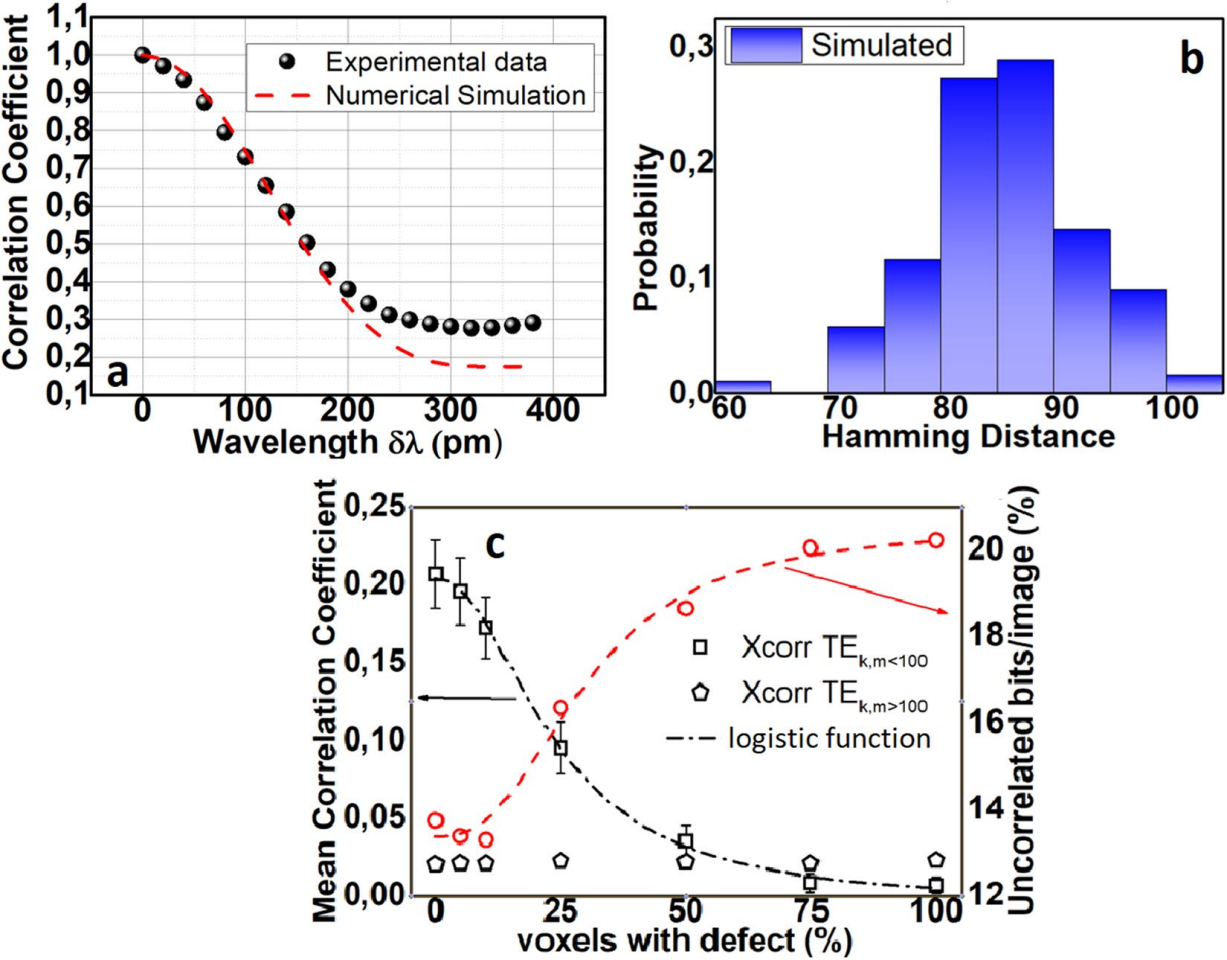
(**a**) Correlation coefficient variation versus the wavelength of the illumination source (**b**) Probability density function of hamming distances for 100 different PUF instances. (**c**) Mean cross correlation (black) for 100 different illumination conditions versus the percentage of output facet defects for 100 TEm, k = 1..10 modes (black square) TEm, k > 100 modes (black-pentagons). A fitting based on a logistic function is assumed in the first case (black slash). The average extractable bits/image were computed in the right y-axis (red-circle) for the low order modes.

Then we assumed an operational scenario where the PUF-core remained constant but the wavelength of the tunable source varied from 1540.0 to 1540.4 m with step Δλ_min_ = 10 pm. In Fig. 7a the correlation coefficient of the first image (λ = 1540 nm) versus all the subsequent images for both experimental and simulated PUFs, is presented. It can be seen that experimental and numerical data notably coincide until Δλ ≈ 200 pm, whereas for higher detuning minor deviation occurs. The experimental results demonstrate an increased plateau (≈0.28) that stems also from the presence of ambient light. In the numerical model, wavelength variations were only linked to the phase accumulated due to propagation, for each mode. More complex effects like variations in the spatial mode distribution versus wavelength or scattering processes were neglected. Furthermore, simulations with increased number of modes but constant maximum phase velocity difference provided similar results to Fig. 7a. Therefore, it can be extracted that similarly to the numerical simulations, the experimental recorded wavelength sensitivity can be mainly attributed to the maximum phase velocity difference of the excited modes. We also employed a probability of cloning scenario where all parameters were kept constant, besides the facet defects. We assumed a single inter-modal distribution, 100 random high-order modes and 100 different output defect arrangements (hypothetical different PUFs). The generated speckles were digitized using random binary technique. In Fig. 7b the probability of hamming distances between code-word pairs is presented. The mean value is 86.2 bit-flips ∼ 33.2%, significant lower compared to Fig. 3b (107 bit-flips ∼ 42%). This deviation stems from the absence of noise effects in the numerical approach. If noise level (Fig. 4b − 13.7 bit flips) is added, then a mean hamming distance of 100 can be estimated which is close to the experimental value of 107.

**Figure 8:**
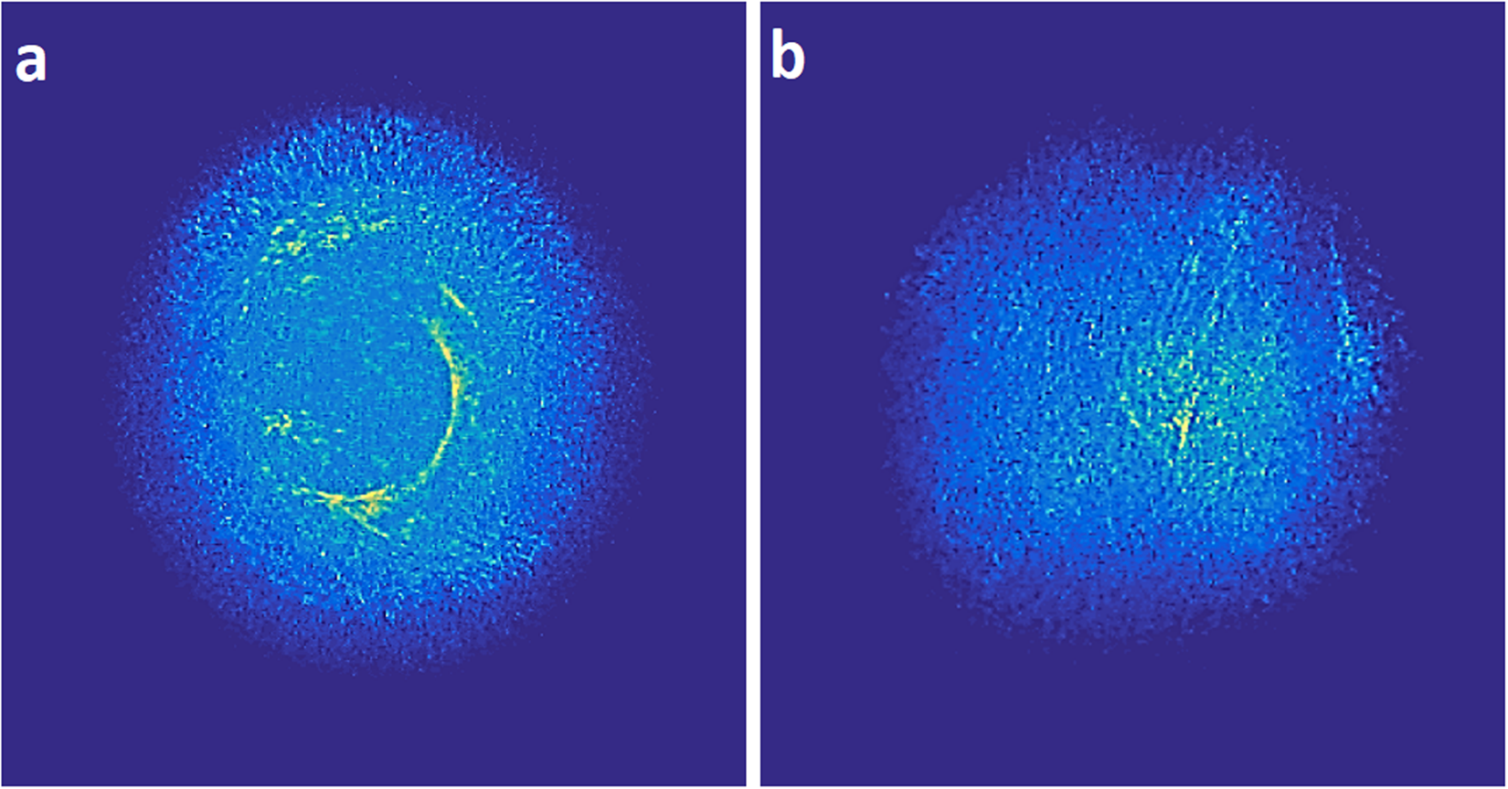
Experimental images of a 10 cm PUF’s output where (**a**) the input facet is highly defective and the output facet is polished and (**b**) vice versa.

The matching of simulation and experimental results evoke the predictive capabilities of the numerical model so as to extract guidelines regarding optimum operational conditions. Towards this direction, we assumed two different initial transverse mode sets. The first consist of consecutive low-order modes (TE_k,m=1…10_), while the second comprised 100 high-order random modes (TE_k,m>100_). For both sets, 100 random inter-modal power distributions were assumed. The correlation coefficient of all corresponding speckle patterns was computed versus the percentage of voxels that contribute to the output facet. It can be seen that the increase of defect’s percentage induces a decrease (Fig. 7c) of the mean correlation coefficient for the low-order modes (black-square) from an initial value of 0.25 to a level lower than 0.05. This result highlights the impact of the output facet in the system’s performance. In particular, low order modes regardless of their power distribution tend to exhibit reduced spatial complexity. This effect is also confirmed by the computation of the percentage of average independent extractable bits per image (methods) presented in Fig. 7c – red circle)^46^. For a constant PUF-camera distance, this metric is directly linked to the entropy of each image and thus to spatial complexity. It can be seen that an increase is present (Fig. 7c red- slash-dot) versus the percentage of defects that can reach a level of ∼20%. Interestingly, when the high-order mode set is used (black-pentagons) the aforementioned trend is not observed. Regardless of the defect’s percentage the cross-correlation remains low <0.05. This can be attributed to the inherent spatial complexity of the high-order modes; whose spatial distribution resembles speckle-like distributions (modal noise). The physical implications of these results are that a fiber-based PUF with highly defective input, evokes high order modes and allows the same performance in terms of image entropy compared to a PUF with highly polished input but defective output. Contrary, physical unclonability is strengthened by the existence of defects at both facets. These numerical results were also experimentally validated, by employing a polymer fiber where one facet was polished using a diamond polishing system. In Fig. 8a,b responses of the PUF were acquired by using the polished facet as input and output respectively. Analysis of datasets similar to the ones used in Fig. 4b,c provided identical performance for both cases, with mean hamming distance of H = 93 ± 15.9 for Fig. 8a and H = 95.7 ± 16.7 for the case of Fig. 8b. Therefore, similar to the numerical simulations, the experimental results confirm that the responses’ spatial complexity follows the complexity imposed by the facet; meaning that one defective facet either at the input or output can scramble modes.

## Discussion

The demonstrated results, regarding unintentional cloning alongside the enhanced system’s sensitivity to external perturbations, solidify the advantages of the proposed PUF, in terms of physical cloning. Therefore, even in the case of a malicious manufacturer, cloning is significantly harder because replication involves two facets and the exact structure of a multi-centimetre long waveguide. In particular, a malicious manufacturer, aiming in physical replicating in-fiber structures, would be obliged to monitor the precise spatial distribution of the optical modes inside the waveguide. This task is not easily accomplished, even if sophisticated high-cost non-invasive techniques, like near field scanning microscopy^43^ or photo-modulation spectroscopy^44^, are employed. Additionally, a simple modification of the PUF specimen that will include a reflective or absorbing coating at the fiber’s surface, can prevent such attacks. Interestingly, even if a malicious manufacturer alleviates all these restrictions and gains complete knowledge over the physical structure of the PUF, he/she would have to utilize sophisticated imprinting techniques^52^ for replicating the facets. These methods exhibit disproportional cost to the cost of the device and drawbacks when used in polymer materials^52^, while the physical replication of in-fiber structures, refractive index variations, cracks, bubbles, bends, etc. impose an ever more stringent problem. Additionally to replication resiliency, fiber’s sensitivity to perturbations could allow system re-configurability and tamper resistance. Meaning that a controlled tension will modify the fiber’s inter-modal power distribution. This is very important for two reasons; it can increase the usability of the PUF, by refreshing the challenge-response space; whereas in case an attacker tries to physically compromise the device, the PUF’s TF will change, eradicating access to the information “stored” within the physical structure. Furthermore, the enhanced sensitivity to mechanical deformations is partially responsible for the slightly decreased robustness of our scheme (Fig. 4d) and is a design factor for prototypes, designated to be used in real-life conditions.

A different attack, consists of extracting the overall TF of the PUF, thus allowing the emulation of its response without any physical replication. In the case of a conventional optical implementation, a spatial light modulator could alter the amplitude and phase of each incoming mode (N_v_ pixels) and an imaging device could monitor the output modes^41,53^, towards replacing the PUF with an emulated wavefront. Although exhaustive TF extraction is not trivial^1^ and is linked to the input wavelength (${N}_{v}=\frac{2\pi {\rm A}}{{\lambda }^{2}}$), this technique can reduce the security offered by optical PUFs^41,53^. Our approach, although radically more resilient to physical replication, is also vulnerable to such an attack^39^ due to the lower TF rank. This will also affect the number of linearly independent challenge-response pairs, if a strong PUF scenario is envisioned. On the other hand, it is worth mentioning that the rank reduction would affect only the necessary mathematical processing, following device characterization. Meaning that the facets’ defects would force the attacker to probe all modes (N_v_) so as to identify the linearly independent mode pairs. It is also worth mentioning, that the rank induced reduction in the challenge-response pairs, does not affect the targeted application, which utilizes the PUF as a single-response authentication token and associates its security features only with physical unclonability.

Finally, the experimental prototype used in this work is based on an off-the-shelve fiber, we chose this configuration due to its cost and availability. It is obvious that the principle of operation can be easily scaled down by using integrated waveguides, thus minimize the mode-filtering process and preserve-enhance fabrication related defects such as surface roughness, impurities etc. Such an endeavour can pave the way for a low-cost, integrated optical PUFs with superior overall performance compared to the state of the art.

## Conclusion

A physical unclonable function based on a solitary optical waveguide is presented. The proposed scheme was evaluated, experimentally and numerically, and found to offer significantly reduced probability of physical cloning, assuming an honest manufacturer, compared to typical optical PUFs. The enhanced performance relies on the simultaneous existence of multiple physical scrambling mechanisms like the facet defects, micro bends and internal fiber defects, rendering also physical cloning attempts by a malicious manufacturer more demanding. Finally, although the demonstrated device is built by bulk optical components the principle of operation can be transferred in a photonic integrated platform, thus enabling significant miniaturization.

## Methods

### Generic Experimental Setup

The fiber itself is a commercially available polymer optical fiber 12 cm long with a step-index core of 980 μm in diameter and 20 μm cladding. The fiber’s facets are cleaved but not polished, while additional defects are generated through a noise-driven friction system. The excitation of the system is being provided by a tunable, single-mode, distributed feedback laser (DFB) emitting in the near infrared (NIR) (λ = 1540 nm, P = 1 mW). The tuning range of the laser extends from 1520 nm to 1570 nm with a minimum wavelength step of 1 pm. The output of the laser is then fed to an erbium doped fiber amplifier (EDFA) that boosts optical power so as to compensate for the elevated losses of the polymer fiber in the target waveband (∼15 dB gain). Light is fed to the POF through simple butt-coupling from a single mode SiO_2_ fiber with numerical aperture of 0.1. The output facet of the POF is imaged through an objective lens with NA = 0.85 and 6 mm focal length to an NIR-camera (vidicon), located 15 cm away from the POF, while a neutral density filter attenuates the received optical power so as to eradicate intensity saturation effects at the camera. The analogue image is digitized through a commercial analogue to digital converter (ADC) and an 8-bit grayscale image with resolution of 340 × 340 pixels is stored. Finally, the recorded images are sent to a personal computer (PC) for post-processing and analysis. For the evaluation of the polished fiber, one facet is cleaved and has been polished using a diamond-based procedure. The source was a HeNe tube emitting at visible (652 nm) followed by a standard high definition camera. The variation of the source and recording device does not affect generality of the investigation, but we have avoided any comparison with previous results through the DFB laser.

### Hashing Techniques

The sparse decompositions $\bar{y}\in {{\mathbb{R}}}^{M}\,$of the images $y\in {{\mathbb{R}}}^{N}\,$were calculated in a known basis $\bar{y}=\Theta y$, where the projection matrices $\Theta \in {{\mathbb{R}}}^{M\times N}$ were constructed via two techniques; the Random and the Gabor Binary techniques. In the former $,\,\Theta =S\times F\times U$, where $U=diag({\{-1,+1\}}^{N})\,$with $Pr[{U}_{ii}=\pm \,1]=0.5$, $F\in {{\mathbb{R}}}^{N\times N}$ the discrete Fourier table and S a matrix containing M random entries with a uniform distribution (0, N). In the latter, every image was processed with a Gabor filter bank of four different orientations, down-sampled and the M = 255 resulting elements (Gabor coefficients) with the highest absolute value were kept. Thereafter, M Gabor Filters, centered at the coordinates of the optimal coefficients, were constructed and converted to one- dimensional arrays, the concatenation of which resulted in the intended matrix. In both procedures, the corresponding results were converted to binary by thresholding the real part of each item by its mean.

### Key Generation Process

For the elimination of errors caused by noise, the core of the key generation process was based on a fuzzy extractor scheme. This maps every hashed response to a unique binary output z and it includes two modes; setup (Fig. 3b) and authentication (Fig. 3c). The former corresponds to the first time that a challenge is applied, whereby the output string is generated along with a set of public helper data, while the latter represents the rerun of the measurement during which the attempt to recreate the same result z is made, by using the helper data produced in the setup mode. An ECC algorithm is used for the detection and the correction of any discrepancies.

### Numerical Model

The core of the numerical model is based on the on the computation of the spatial profile and effective refractive index of supported modes in a commercial polymer optical fiber, with 980 μm core and 20 μm cladding. In order to simulate the random defects at the input facet a matrix S_1_ is assumed with size equal to the number of wavelength sized voxels of the facet. Its elements exhibit a Gaussian distribution with mean value equal to zero and standard deviation equal to one. The spatial profile of each mode (M_k_) is multiplied with S_1_ and the integral of this product is normalized to the integral of M_k_ assuming zero facet losses. Through this process, the fractional power for each mode during launching is computed. The excited modes are chosen through a uniform random process, thus we can control both the type and power of transverse modes. A typical example is presented in Fig. 6a where we have assumed that facet defects have evoked 100 random high order TE_m,k_ with m, k >100 exhibiting a normal power distribution. For the sake of simplicity, we assumed multi-mode propagation through an ideal fiber with length (L) without taking into consideration mode-coupling effects. This simplification does not affect generality; the random nature of the initial modal distribution is considered to model also potential in-fiber mode-coupling effects. Therefore, in-fiber propagation results in a phase shift for each mode that is linked to its unique phase velocity. After propagation the modes interact with the output facet, which according to the theoretical analysis, acts as a typical diffuser. Therefore, a complex matrix S_2_ is computed where the real part corresponds to scattering induced amplitude attenuation and follows a normal distribution with zero mean and sigma of one. The imaginary part corresponds to a random phase shift induced by a typical optical diffuser, with sigma of 2π and mean value of zero. The near-field at the PUF’s output facet is computed by $Real({S}_{2})\cdot E\cdot {e}^{-j\cdot Imag({S}_{2})}$, where E represented the complex optical field illuminating the output facet (see Fig. 5b). For the sake of simplicity, we neglected optical feedback evoked through residual facet reflectivity.

The approach used for the computation of the speckle image at a hypothetical imaging device consists of the following steps: The distance between the fiber’s facet and the camera is denoted D_x_. The surface of the facet is divided in wavelength sized voxels that can be considered independent point sources. The coordinates of the i_th_ point source at end facet of the fiber are (x_i_, y_i_) while the coordinates of the j_th_ pixel of the imaging module array are (x_j_, y_j_). The camera array consists of L_c_ rows and H_c_ columns. The distance between the i_th_ point source and j_th_ pixel is: ${d}_{ij}=\sqrt{{({x}_{i}-{x}_{j})}^{2}+{({y}_{i}-{y}_{j})}^{2}+D{x}^{2}}.$ The total value of the incident field at the j_th_ pixels of the imaging module array is then given by: ${E}_{j}=\sum _{i=0}^{{N}^{2}}{E}_{i}\cdot {e}^{j\frac{2\pi }{\lambda }{d}_{ij}},$ where E_i_ is the complex field exiting the i_th_ point source. By varying the distance D_x_ we can manipulate the average speckle size (grain). The average speckle size, or equivalently the ratio pixels/speckle can be theoretically predicted through diffraction theory: $S=\frac{\lambda \cdot {D}_{x}}{R}$. For the extraction of the average grain size in the generated simulation images we also used the same methodology used when treating experimental speckles. We computed the autocorrelation function for each image followed by mathematical fitting and full-width at half-maximum extraction, which corresponds to the average speckle size in pixels/speckle.

## Electronic supplementary material

Supplementary information
