# Supplementary material for: Physical Unclonable Function based on a Multi-Mode Optical Waveguide
Source: Sci Rep. 2018 Jun 25;8:9653. doi: 10.1038/s41598-018-28008-6 (PMC6018550; doi:10.1038/s41598-018-28008-6)
Supplement: Supplementary file 1 — Supplementary information [file 41598_2018_28008_MOESM1_ESM.docx]

Physical Unclonable Function based on a Multi-Mode Optical Waveguide

Charis Mesaritakis^a*^, Marialena Akriotou^b^ , Alexandros Kapsalis^a^, Evaggelos Grivas^a^, Charidimos Chaintoutis^b^, Thomas Nikas^b^, Dimitris Syvridis^b^

1. Eulambia Advanced Technologies Ltd. Ag. Ioannou 24, 15342-Athens Greece
2. Department of Informatics & Telecommunications, National and Kapodistrian University of Athens, Panepistimiopolis Ilisia 15784, Athens, Greece

email*: [charis.mesaritakis@eulambia.com](mailto:charis.mesaritakis@eulambia.com)

**Supplementary Information**

***Security Framework for PUF***

The physical component of the system is produced via a process called Create (fig.1 for a complete overview of the scheme). This process is carried out by the manufacturer, who chooses a fixed creation parameter α_CR_, that corresponds to all the controllable conditions of the fabrication procedure. In our case, α_CR_ contains the industrial construction of the plastic optical fiber, along with its preselected fixed length (i.e. 12cm). On the other hand, the uniqueness of the component derives from the random variations of the production (namely the creation noise), which in the current study are the inherent imperfections of the fiber, as well as, the unsystematic fashion in which the grooves are created at its facet.


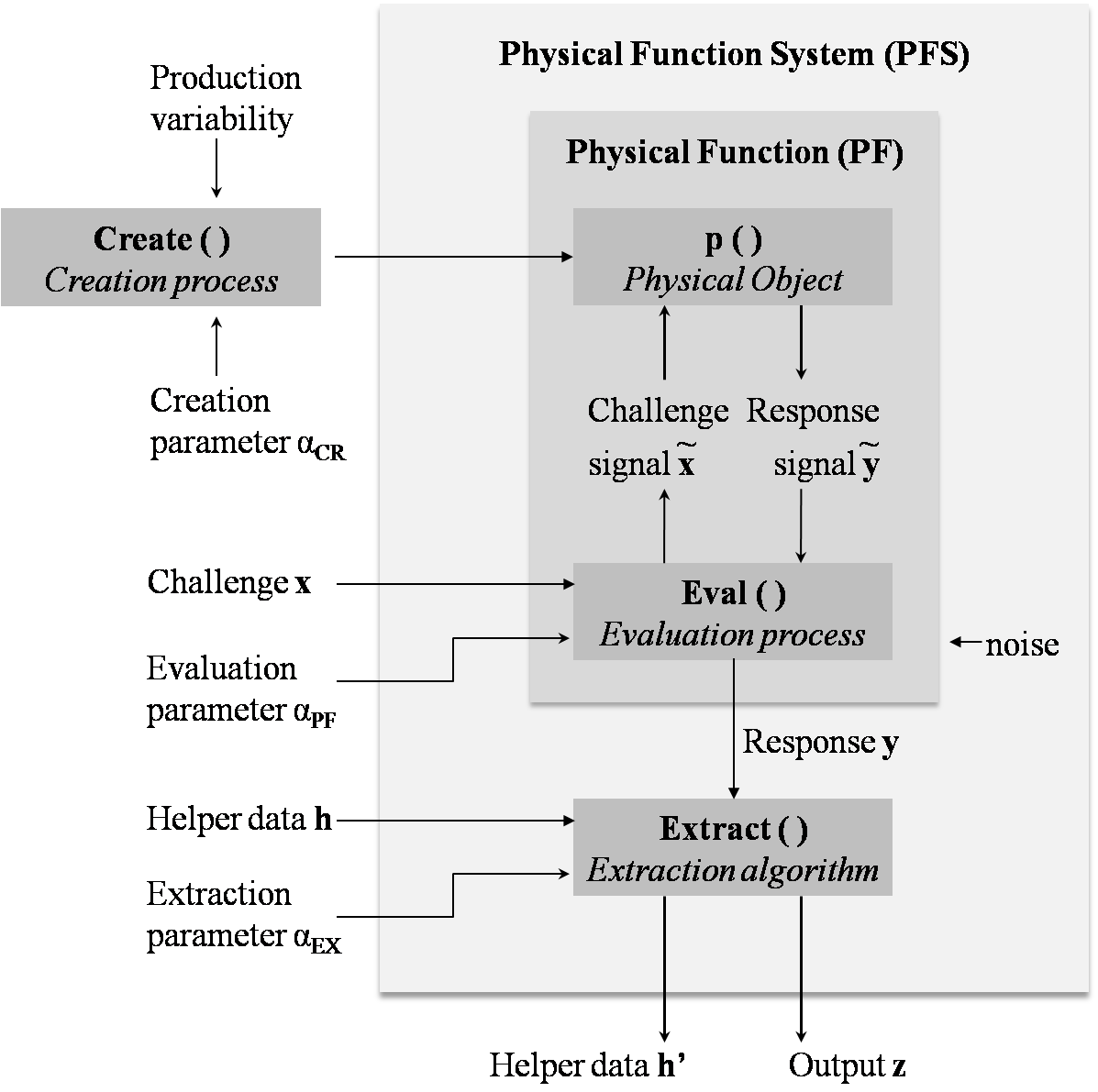


Fig.1 The generic security framework of a PUF system realization.

The main body of the system, the so-called physical function (PF), consists of the component p, along with a procedure Eval; when a challenge x is applied to the PF, its excited object will result in an analog signal response$\tilde{y}$, which is converted to its digital representation y and measured (i.e. the image recorded) through the Eval procedure. The conversion and measurement settings of the procedure are denoted as the evaluation parameter α_PF_, which is specified by the manufacturer. In optical-fiber-PUFs, the Eval process and the α_PF_ parameter correspond to the detection apparatus used and its resolution respectively.

It should be pointed out that the execution of the Create and Eval procedures gives rise to two different types of noise. The former leads to the desirable creation noise, which is responsible for the true randomness of the instantiations, while the latter causes the undesirable observation noise, the effects of which can be negated up to a certain point by the algorithm Extract.

In other words, the primary purpose of the Extract algorithm is the correction of the slight dissimilarities induced by noise and observed amongst different y recordings that derive from one single challenge x (fig.2). This can be accomplished by means of a fuzzy commitment scheme along with an EC algorithm. In essence, the fuzzy commitment scheme maps the different responses y to a unique output bit-string z, based on a fixed extractor parameter α_EX_ (i.e. the M, l, d values of the ECC). Therefore, the Extract algorithm is always carried out in two different modes, the setup mode (fig.1d) and the authentication mode (fig.1e). The former corresponds to the first time that a challenge is applied, whereby the output z is generated, along with a set of appropriate helper data h. The latter represents the rerun of the measurement, during which the attempt to recreate the same result z is made, by using the helper data produced in the setup phase. At the same time, the ECC algorithm is utilized for the detection and the correction of all the possible z results’ discrepancies, leading to the decrease of their in-between bit error rate.


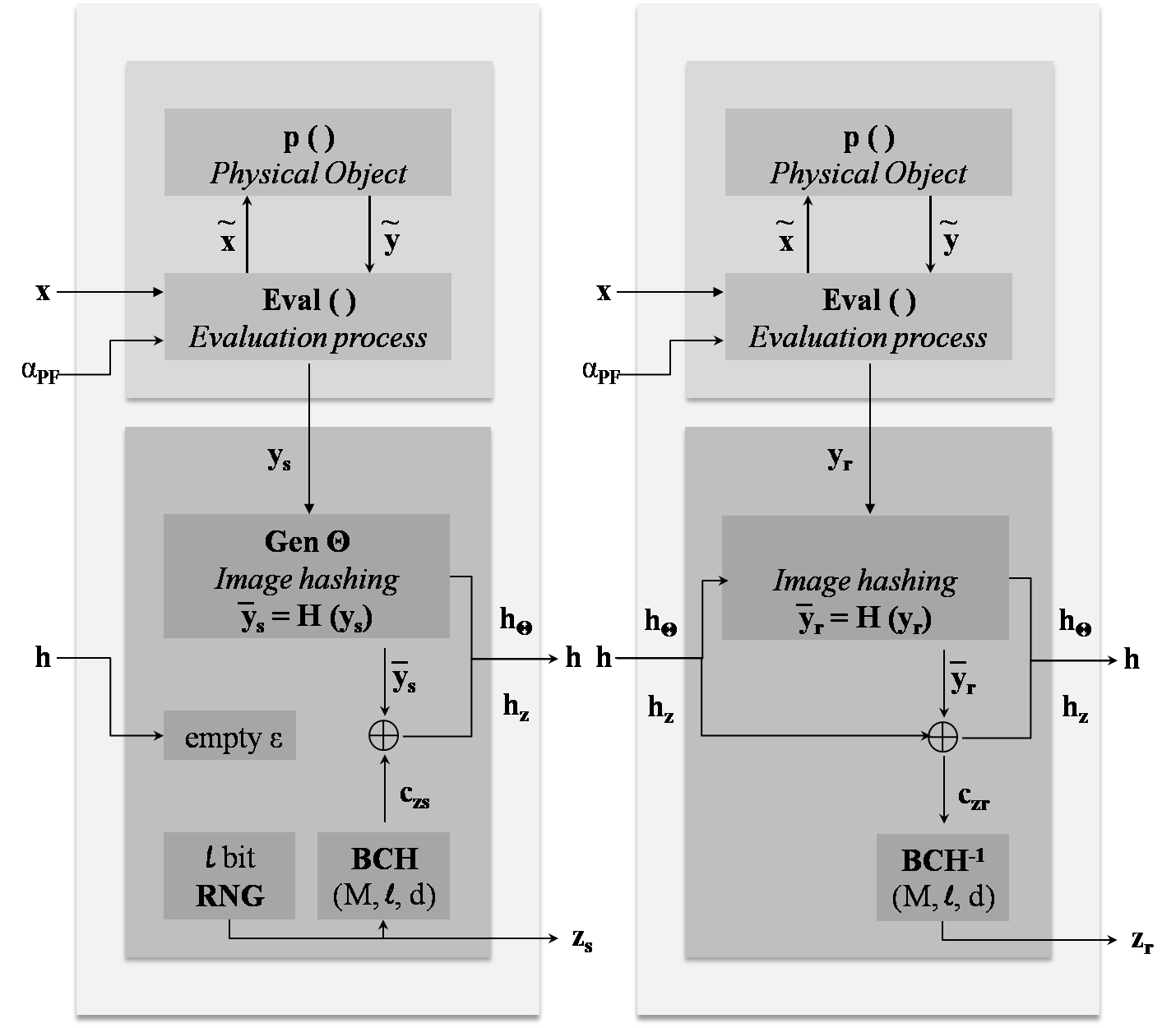


Fig.2 The fuzzy extractor scheme utilized for the extraction of errorless responses

Generally, the Extract process as described in the previous paragraph can be put into practice without any additional steps for the majority of the existing PUF system implementations. In our case however, the responses y obtained were the pixel intensity matrices of the speckle images, recorded in a high resolution (denoted below as N_1_xN_2_). The large dimensions of the data, along with the fuzzy extractor’s requirement for a binary output, necessitates the introduction of an extra step in the procedure, aimed firstly in the reduction of the matrices’ size and secondly in the subsequent quantization of the results.

In that respect, the procedure integrated for fulfilling the aforementioned needs was a compressive sensing-based hashing algorithm, which can be summarized through the following relation:

$\overline{y}=H\left(y\right)=sign\left(\Theta y\right)$ (1)

, where y is the PUF response converted to a one- dimensional array of size N = N_1_xN_2_ (i.e.$y\in\mathbb{R}^{N}$) and $\bar{y}$ the corresponding resultant hashed bit-string of length M ≤ N (i.e.$\bar{y}\in\mathbb{R}^{M}$). The compression is essentially achieved by sensing the array y with a $\mathbb{R}^{\mathrm{MxN}}$table Θ, namely the sensing matrix, while the subsequent quantization of the result derives from the function sign, which is defined as: $sign\left(\bar{y}\left(\lambda\right)\right)=\left\{\begin{matrix} \begin{matrix} 1 & for & \lambda>mean(\bar{y}) \end{matrix} \\ \begin{matrix} 0 & for & \lambda<mean(\bar{y}) \end{matrix} \end{matrix}\right.$ (2)

To sum up, the Extract algorithm that was practically employed is presented in *Fig. 1d-e*. Its first schematic represents the setup mode of the system during which, a challenge x is applied for the first time. The corresponding bit-string $\bar{y}_{s}$for this challenge is extracted according to equation (1), while the sensing matrix Θ used is stored in a helper data file h_Θ._ Simultaneously, a random bit-string z_s_ of length l ≤ M is independently generated and subsequently encoded through a BCH algorithm into a bit-string cz_s_ of length M. Then, a second helper data file h_z_ is stored, containing the result of the XOR operation between the bit-strings $\bar{y}_{s}$ and cz_s_. The second schematic illustrates the reconstruction mode of the system: a response y_r_ is obtained under the same challenge as in the setup mode. The corresponding hashed bit-string $\bar{y}_{r}$ is calculated through the helper data h_Θ_ and the XOR between $\bar{y}_{r}$ and h_z_ helper data is extracted, resulting in a cz_r_ bit-string. The latter is decoded through the inverse BCH code and a bit-string z_r_ is finally concluded.

It is noteworthy that the selected BCH parameters (M, l, d) play a significant role upon the effectiveness of the Extract procedure described above. As the ECC theory dictates, when l is the number of information bits encrypted in a code - message of length M = 2^k^ – 1, and d corresponds to the minimum number of bits that differ between two codes, the error correction capability of a BCH algorithm is given by the relation t = (d - 1) / 2. Essentially, the employed algorithm can detect and correct up to t errors through the redundant bits added during the encoding process of the setup mode. The length l has an inversely proportional relation to the available overhead and the t capability of the code; given a fixed value of M, increasing l results in the correction of fewer bit-flip-errors, and subsequently leads to the reduction of the responses’ number that is ultimately mapped to one single output z.

***Random Binary extraction procedure***

The Random Binary technique refers to the non-adaptive approach, during which the entries of the sensing matrix are selected in advance according to a Bernoulli, a Gaussian or a sub-Gaussian distribution. The restricted isometry property of such matrices enables the compact representation of high-dimensional sparse signals with only a small amount of loss or corruption, preserving their in-between Euclidean distances and securing their robust reconstruction. Nevertheless, the sensing matrix used in our case is equal to Θ = S*F*U. U represents a diagonal random table (NxN), containing only the values ± 1 with P_r_ [U_ii_ = 1] = P_r_ [U_ii_ = -1] = 0.5, and F the discrete Fourier table of (NxN) dimensions. Finally, S symbolizes a matrix containing M entries randomly chosen from a uniform distribution (0, N); those were the indices of the elements, being extracted to constitute the corresponding hashed bit-string. The result of the above procedure was converted to binary by thresholding the real part of each item by the mean. This alternative algorithm, has been proven to manifest a similar behaviour to the conventional technique with a full Gaussian distribution. However, it can provide a faster extraction of the hashes, performing only O(NlogN) computations compared to the O(MN) of the latter.

***Gabor Binary extraction procedure***

The Gabor Binary method, on the other hand, is the adaptive approach of the hashing procedure in which the entries of the sensing matrix Θ are reliant on a previously acquired image. According to the relevant theory, a two-dimensional Gabor filter is defined as the multiplication product of a plane wave with a 2D Gaussian envelope. It is a complex function, whose imaginary component is given by the following relation:

$$g_{\nu_{0},\theta,\sigma}\left( x,y \right)=\frac{1}{2\pi\sigma^{2}}exp\left( -\frac{x^{'2}+y^{'2}}{2\sigma^{2}} \right)sin\left( \nu_{0}x^{'} \right)$$

For the above equation, it holds that$x^{'}=xcos\left( \theta\right)+ysin\left( \theta\right)$and$y^{'}=-xsin\left( \theta\right)+ycos\left( \theta\right)$, with θ the orientation of the stripes created. σ is the standard deviation of the Gaussian window centered at (0,0), while ν_0_ the spatial frequency of the sine factor.

The values of the Gabor parameters used in our case were, ν_0_ = π/3, σ = 10 and θ = π*f/F, with 0 ≤ f ≤ F and F = 4 respectively. Consequently, a Gabor filter bank with four different orientations was created, each of which was applied to every image separately. Thereafter, every resultant matrix was down-sampled by dividing it into (30x30) squares and keeping their last element. As a result, 121*4 = 484 Gabor coefficients were obtained, from which the M ≤ 484 (i.e. M = 255) elements with the highest absolute value were kept for use. Then, 255 Gabor Filters, centered at the coordinates of these optimal coefficients, were constructed and converted to one- dimensional arrays, the concatenation of which resulted in the intended sensing matrix$\Theta\in\mathbb{R}^{\mathrm{MxN}}$. Finally, the hashed bit-string emerged from the multiplication of the sensing matrix with the image and the conversion of the result to binary, which was carried out as in the Random Binary method.
